# Pulsed Electromagnetic Field Exposure Attenuates Ultraviolet B-Induced Dermal Collagen Loss in Association with A2A Adenosine Receptor Signaling

**DOI:** 10.3390/ijms27177904

**Published:** 2026-09-04

**Authors:** Kyung-A Byun, Jae Ik Lee, Seyeon Oh, So Eun Kim, Suk Bae Seo, Kuk Hui Son, Kyunghee Byun

**Affiliations:** 1 Department of Anatomy & Cell Biology, College of Medicine, Gachon University, Incheon 21936, Republic of Korea; 2 LIBON Inc., Incheon 22006, Republic of Korea; 3 Functional Cellular Networks Laboratory, Lee Gil Ya Cancer and Diabetes Institute, Gachon University, Incheon 21999, Republic of Korea; 4 Department of Thoracic and Cardiovascular Surgery, International Healthcare Center, Seoul National University Bundang Hospital, Seongnam 13620, Republic of Korea; 5 Cheongdam Ruby Clinic, Seoul 06009, Republic of Korea; 6 SeoAh Song Dermatologic Clinic, Seoul 05557, Republic of Korea; 7 Department of Thoracic and Cardiovascular Surgery, Gachon University Gil Medical Center, Gachon University, Incheon 21565, Republic of Korea; 8 Department of Health Sciences and Technology, Gachon Advanced Institute for Health & Sciences and Technology (GAIHST), Gachon University, Incheon 21999, Republic of Korea

**Keywords:** pulsed electromagnetic field, photoaging, collagen, A2A adenosine receptor, cyclic adenosine monophosphate, protein kinase A, NLRP3 phosphorylation, interleukin-1β, nuclear factor-κB, matrix metalloproteinase

## Abstract

Ultraviolet (UV) exposure accelerates skin aging through wavelength-dependent effects. Ultraviolet A (UVA) penetrates relatively deeply into the dermis and promotes oxidative stress and extracellular-matrix remodeling, whereas ultraviolet B (UVB) is absorbed predominantly in the epidermis and produces direct DNA damage and inflammation; nevertheless, UVB can also alter dermal fibroblast matrix metabolism in experimental models. The present study specifically used UVB irradiation (peak wavelength, 306 nm) to examine inflammatory collagen loss. UVB-induced cellular injury activates the NOD-like receptor family pyrin domain-containing 3 (NLRP3) inflammasome, leading to caspase-1-dependent maturation of interleukin-1β (IL-1β), activation of nuclear factor-κB (NF-κB), and matrix metalloproteinase (MMP)-associated collagen degradation. Pulsed electromagnetic field (PEMF) stimulation is a non-optical biophysical modality that may modulate inflammatory and matrix responses. Here, we characterized the effects of PEMF exposure using Corefacial on UVB-induced dermal collagen loss in association with the A2A adenosine receptor (A2AAR)/cyclic adenosine monophosphate (cAMP)/protein kinase A (PKA) signaling pathway. In UVB-exposed fibroblasts, PEMF restored A2AAR and PKA protein levels, cAMP levels, and the pNLRP3/NLRP3 ratio, similar to the effects of the A2AAR agonist CGS-21680. PEMF also reduced apoptosis-associated speck-like protein containing a caspase-recruitment domain (ASC), caspase-1 activation markers, IL-1β secretion, NF-κB nuclear translocation, and MMP2/MMP3/MMP9 expression and increased the levels of collagen I and collagen III. In the in vivo UVB model, PEMF similarly increased A2AAR and PKA protein levels, cAMP levels, and the pNLRP3/NLRP3 ratio, while attenuating IL-1β/NF-κB/MMP-associated responses and preserving dermal collagen. These findings are consistent with, but do not prove, involvement of A2AAR/cAMP/PKA-related signaling and inhibitory NLRP3 phosphorylation in the PEMF response. These results should be interpreted as evidence from a UVB-specific experimental model rather than a comprehensive model of solar photoaging.

## 1. Introduction

Solar ultraviolet radiation comprises ultraviolet (UV)A (315–400 nm), UVB (280–315 nm), and UVC (100–280 nm); terrestrial UVC is largely filtered by the atmosphere. UVA constitutes most solar UV reaching the skin and penetrates more deeply into the dermis, where chromophore-mediated photosensitization and reactive oxygen species contribute to fibroblast dysfunction and extracellular-matrix remodeling. UVB is absorbed mainly within the epidermis and is particularly effective at producing cyclobutane pyrimidine dimers, erythema, keratinocyte injury, and inflammatory signaling. Although substantially less UVB reaches the dermis than UVA, UVB can influence dermal matrix homeostasis through epidermal–dermal signaling and can directly alter cultured dermal fibroblasts when applied experimentally [1,2,3,4]. These wavelength-dependent differences are essential when interpreting photoaging models.

Both UVA and UVB contribute to cutaneous photoaging, but they do not constitute interchangeable experimental exposures. UVA-centered models are especially relevant to chronic oxidative injury in dermal fibroblasts, whereas UVB-centered models are commonly used to study direct DNA damage, epidermal inflammation, and downstream collagen/matrix metalloproteinase (MMP) changes [1,2,3,4].

The NOD-like receptor family pyrin domain-containing 3 (NLRP3) inflammasome links UV-induced cellular injury to inflammatory matrix degradation [5,6,7]. Activation of the NLRP3 inflammasome, comprising NLRP3, apoptosis-associated speck-like protein containing a caspase-recruitment domain (ASC), and pro-caspase-1, promotes caspase-1-dependent maturation of pro-interleukin-1β (pro-IL-1β) into active interleukin-1β (IL-1β) [7]. UVB-induced nuclear DNA damage increases NLRP3 expression and IL-1β production in human keratinocytes, whereas NLRP3 knockdown reduces UVB-induced IL-1β as well as IL-1α, IL-6, tumor necrosis factor-α (TNF-α), and prostaglandin E2 [5]. NLRP3 also recognizes UVB-damaged mitochondria, whereas alternative autophagy limits this response by clearing damaged mitochondria [6]. MAPK and NF-κB, downstream of IL-1β signaling, promote MMP induction and suppress anabolic ECM repair programs, providing a plausible route for accelerated collagen loss by UV-triggered inflammasome activation [8,9].

Pulsed electromagnetic field (PEMF) stimulation is a non-optical biophysical modality that uses time-varying electromagnetic fields to alter cellular behavior without relying on chromophore-dependent light absorption [10,11,12,13,14]. PEMF increases collagen production in bone marrow fibroblasts, alters collagen synthesis in rat skin, increases collagen fiber deposition in diabetic wounds, enhances collagen gene and protein expression in tendon cells, and up-regulates intervertebral disc-cell matrix synthesis through bone morphogenetic protein-dependent mechanisms [10,11,12,13,14]. Musculoskeletal studies show that PEMF affects chondrocyte proliferation, proteoglycan metabolism, and cartilage matrix homeostasis [15,16,17]. Thus, although matrix-producing cells respond to PEMF, it is not known whether it protects UV-damaged skin through an anti-inflammatory pathway [10,11,12,13,14,15,16,17].

Adenosine receptor signaling could connect PEMF exposure to inflammasome regulation [18,19,20]; electromagnetic field exposure increases A2A and A3 adenosine receptor mRNA, protein abundance, receptor density, and cyclic adenosine monophosphate (cAMP)-linked functionality in osteoarthritic synovial fibroblasts and related joint cells [18,19,20]. The A2A adenosine receptor (A2AAR) is a stimulatory G-protein-coupled receptor that increases intracellular cAMP and activates protein kinase A (PKA) [18,19,20]. Signaling by cAMP/PKA inhibits NLRP3 inflammasome activity, and PKA-mediated phosphorylation of human NLRP3 at serine 295 is linked to impaired ASC oligomerization and reduced inflammasome assembly [21,22,23,24]. Adenosine-dependent A2AAR signaling is also associated with suppression of NLRP3/caspase-1/IL-1β activation in THP-1 cells [25].

These observations led us to hypothesize that PEMF exposure with Corefacial would attenuate UVB-induced dermal collagen loss in association with restoration of A2AAR/cAMP/PKA signaling and increased levels of phosphorylated NLRP3 (pNLRP3). To test this hypothesis, we characterized A2AAR/cAMP/PKA/pNLRP3-related markers, the NLRP3 inflammasome-associated pathway, IL-1β/NF-κB/MMP signaling, and collagen abundance in UVB-exposed fibroblasts and in a UVB-induced animal model.

## 2. Results

### 2.1. PEMF Restores Markers Associated with A2AAR/cAMP/PKA/pNLRP3 Signaling in UVB-Exposed Fibroblasts

**Figure 1:**
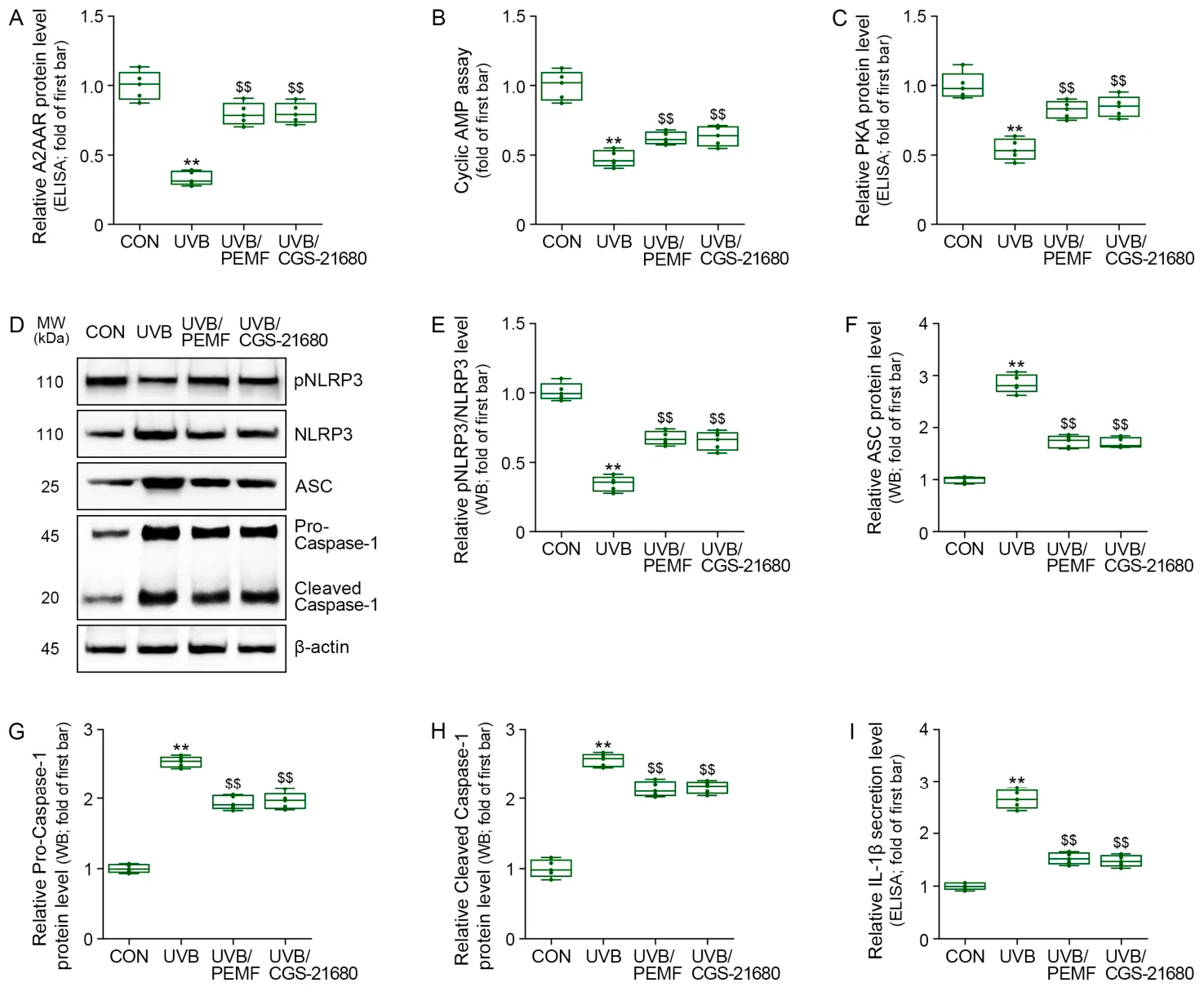
PEMF treatment restores A2AAR/cAMP/PKA/pNLRP3-related markers in UVB-exposed fibroblasts. (**A**) A2AAR protein expression level. (**B**) Intracellular cAMP level. (**C**) PKA protein level. (**D**) Representative Western blot of phosphorylated NLRP3, total NLRP3, ASC, pro-caspase-1, cleaved caspase-1, and β-actin. (**E**–**H**) Quantitative Western blot results for pNLRP3/NLRP3, ASC, pro-caspase-1, and cleaved caspase-1. (**I**) Relative IL-1β secretion. In all quantitative panels, groups from left to right represent CON, UVB, UVB/PEMF, and UVB/CGS-21680. Statistical comparisons were performed using the Mann–Whitney U test. **, *p* < 0.01, vs. CON; $$, *p* < 0.01, vs. UVB. A2AAR, adenosine A2A receptor; ASC, apoptosis-associated speck-like protein containing a CARD; NLRP3, NOD-like receptor pyrin domain-containing protein 3; PEMF, pulsed electromagnetic field; PKA, protein kinase A.

We analyzed A2AAR/cAMP/PKA/pNLRP3-related markers in four groups of fibroblasts: untreated control, UVB-treated control, UVB/PEMF-treated, and UVB/A2AAR agonist (CGS-21680)-treated, with the latter serving as a pharmacologic positive control for A2AAR activation. Compared with untreated control fibroblasts, UVB exposure reduced A2AAR and PKA protein levels, cAMP levels, and the phosphorylated-to-total NLRP3 ratio (pNLRP3/NLRP3). In UVB-exposed fibroblasts, PEMF treatment increased A2AAR, cAMP, and PKA levels and the pNLRP3/NLRP3 ratio. CGS-21680 produced changes in the same direction in UVB-treated fibroblasts compared with UVB-treated controls (Figure 1A–E).

### 2.2. PEMF Suppresses Inflammasome Activation, NF-κB Nuclear Translocation, and MMP Expression and Restores Collagen in UVB-Exposed Fibroblasts

Western blot analysis of UVB-exposed fibroblasts showed increased NLRP3 inflammasome-associated markers, including ASC, pro-caspase-1, and cleaved caspase-1, as well as increased IL-1β secretion. PEMF or CGS-21680 reduced these markers in UVB-exposed fibroblasts (Figure 1D–I).

**Figure 2:**
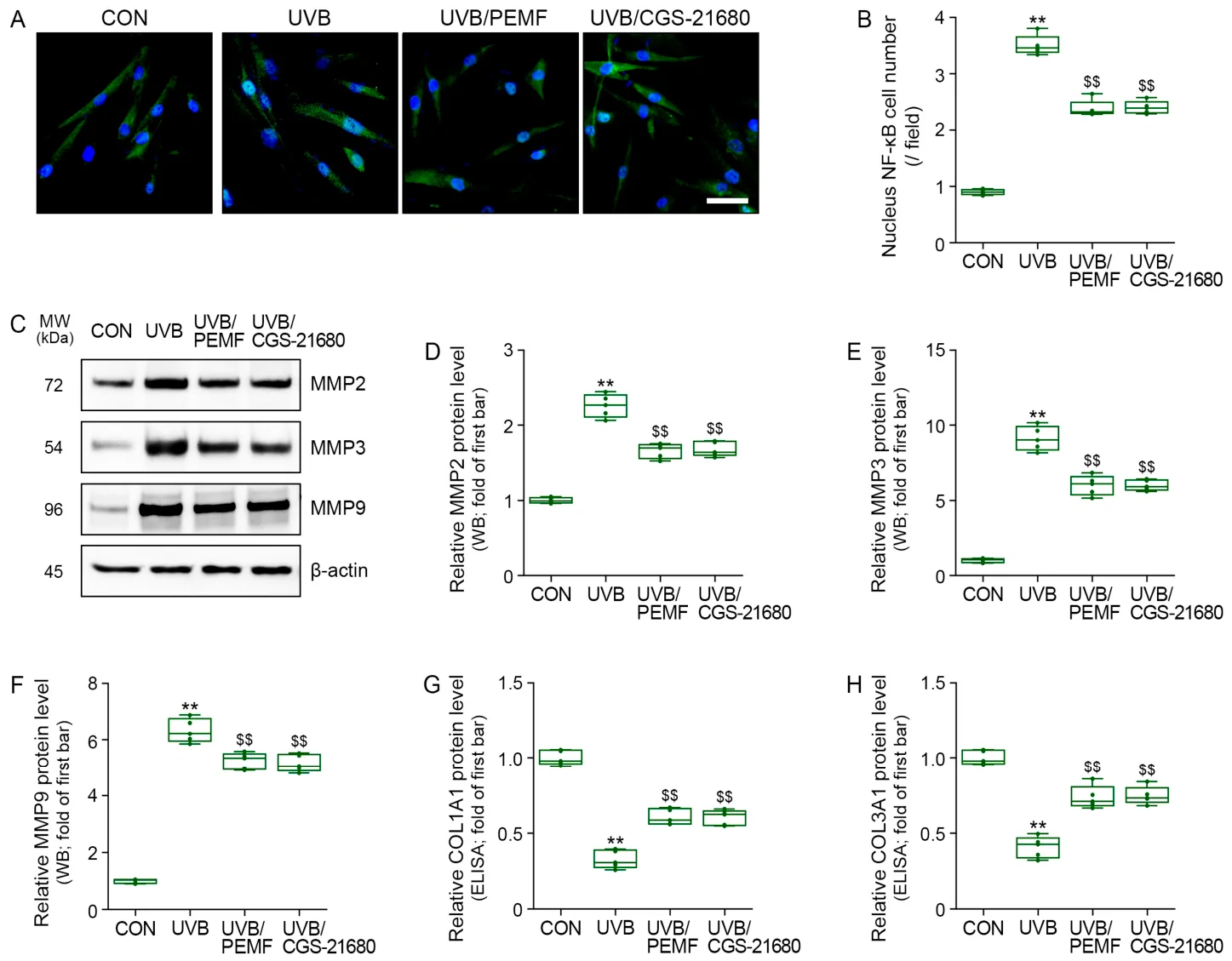
PEMF treatment suppresses inflammatory responses and collagen loss in UVB-exposed fibroblasts. (**A**) Representative immunocytochemical images of NF-κB localization. NF-κB is shown in green, and nuclei are counterstained with DAPI (blue). Scale bar = 50 μm. (**B**) Quantification of cells with NF-κB-positive nuclei. (**C**) Representative Western blot images of MMP2, MMP3, MMP9, and β-actin. (**D**–**F**) Quantitative Western blot results for MMP2, MMP3, and MMP9 expression. (**G**,**H**) Relative collagen I and collagen III protein levels. In all quantitative panels, groups from left to right represent CON, UVB, UVB/PEMF, and UVB/CGS-21680. Statistical comparisons were performed using the Mann–Whitney U test. **, *p* < 0.01, vs. CON; $$, *p* < 0.01, vs. UVB. MMP, matrix metalloproteinase; PEMF, pulsed electromagnetic field.

Immunocytochemistry showed that PEMF reduced UVB-induced nuclear localization of NF-κB (Figure 2A,B). UVB exposure also increased MMP2, MMP3, and MMP9 expression, whereas PEMF or CGS-21680 reduced their expression (Figure 2C–F). Collagen I and collagen III abundance decreased after UVB exposure and increased after PEMF or CGS-21680 treatment (Figure 2G,H). The similar direction of the PEMF and CGS-21680 responses is consistent with, but does not prove, involvement of A2AAR-related signaling.

### 2.3. PEMF Restores A2AAR/cAMP/PKA/pNLRP3-Related Markers in UVB-Treated Skin

**Figure 3:**
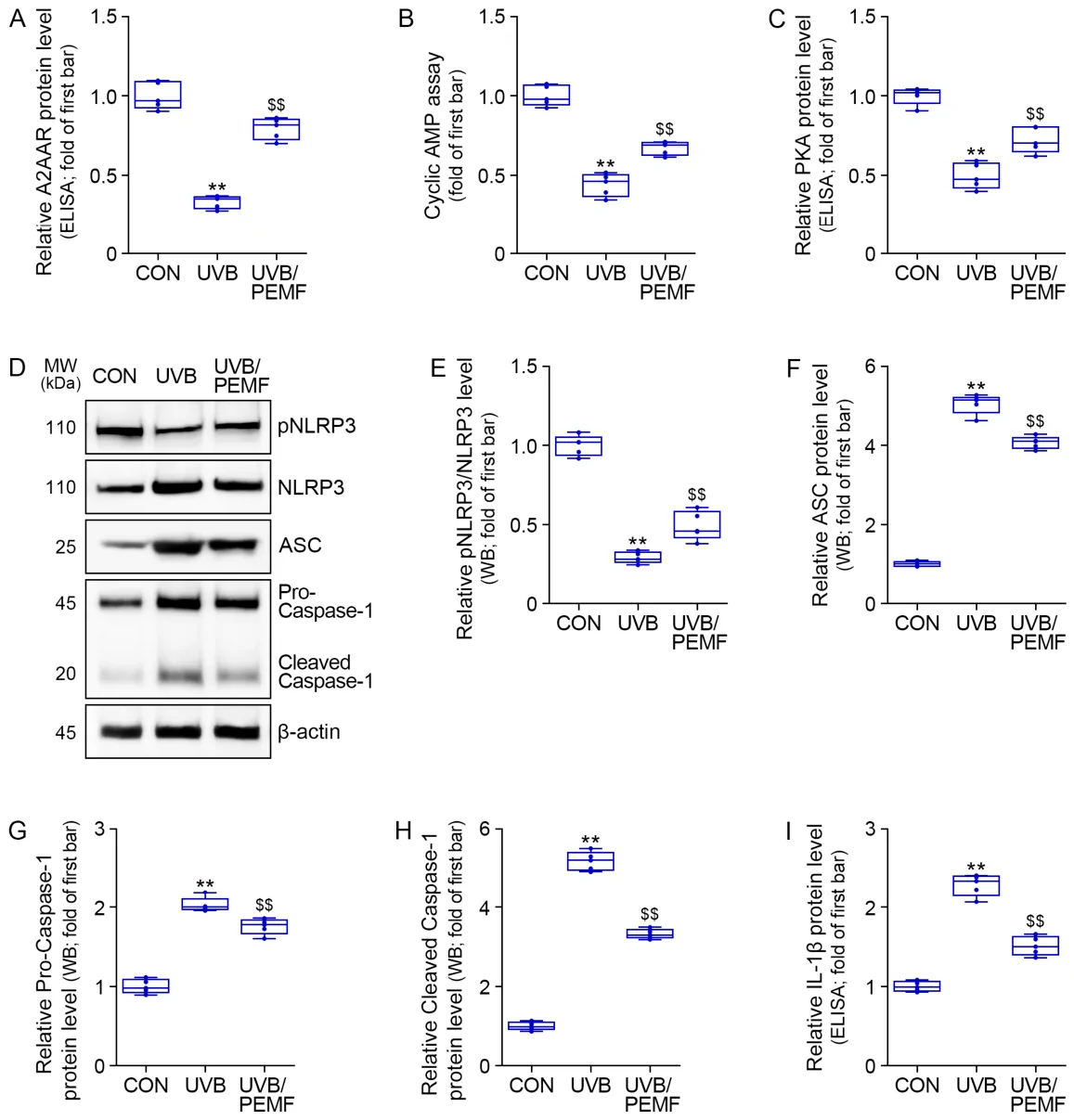
PEMF treatment restores A2AAR/cAMP/PKA/pNLRP3-related markers in a UVB-induced animal model. (**A**) A2AAR protein expression level. (**B**) Intracellular cAMP level. (**C**) PKA protein level. (**D**) Representative Western blot of phosphorylated NLRP3, total NLRP3, ASC, pro-caspase-1, cleaved caspase-1, and β-actin. (**E**–**H**) Quantitative Western blot results for pNLRP3/NLRP3, ASC, pro-caspase-1, and cleaved caspase-1. (**I**) Relative IL-1β protein level. In all quantitative panels, groups from left to right represent CON, UVB, and UVB/PEMF. Statistical comparisons were performed using the Mann–Whitney U test. **, *p* < 0.01, vs. CON; $$, *p* < 0.01, vs. UVB. A2AAR, adenosine A2A receptor; ASC, apoptosis-associated speck-like protein containing a CARD; NLRP3, NOD-like receptor pyrin domain-containing protein 3; PEMF, pulsed electromagnetic field; PKA, protein kinase A.

In the in vivo model, UVB exposure reduced A2AAR and PKA protein levels, cAMP levels, and the pNLRP3/NLRP3 ratio compared with untreated control skin. In UVB-exposed animals, PEMF treatment increased these markers (Figure 3A–E). UVB exposure also increased ASC, pro-caspase-1, cleaved caspase-1, and IL-1β levels, whereas these changes were attenuated by PEMF treatment (Figure 3D–I).

### 2.4. PEMF Suppresses IL-1β/NF-κB/MMP Signaling and Improves Collagen Histology In Vivo

**Figure 4:**
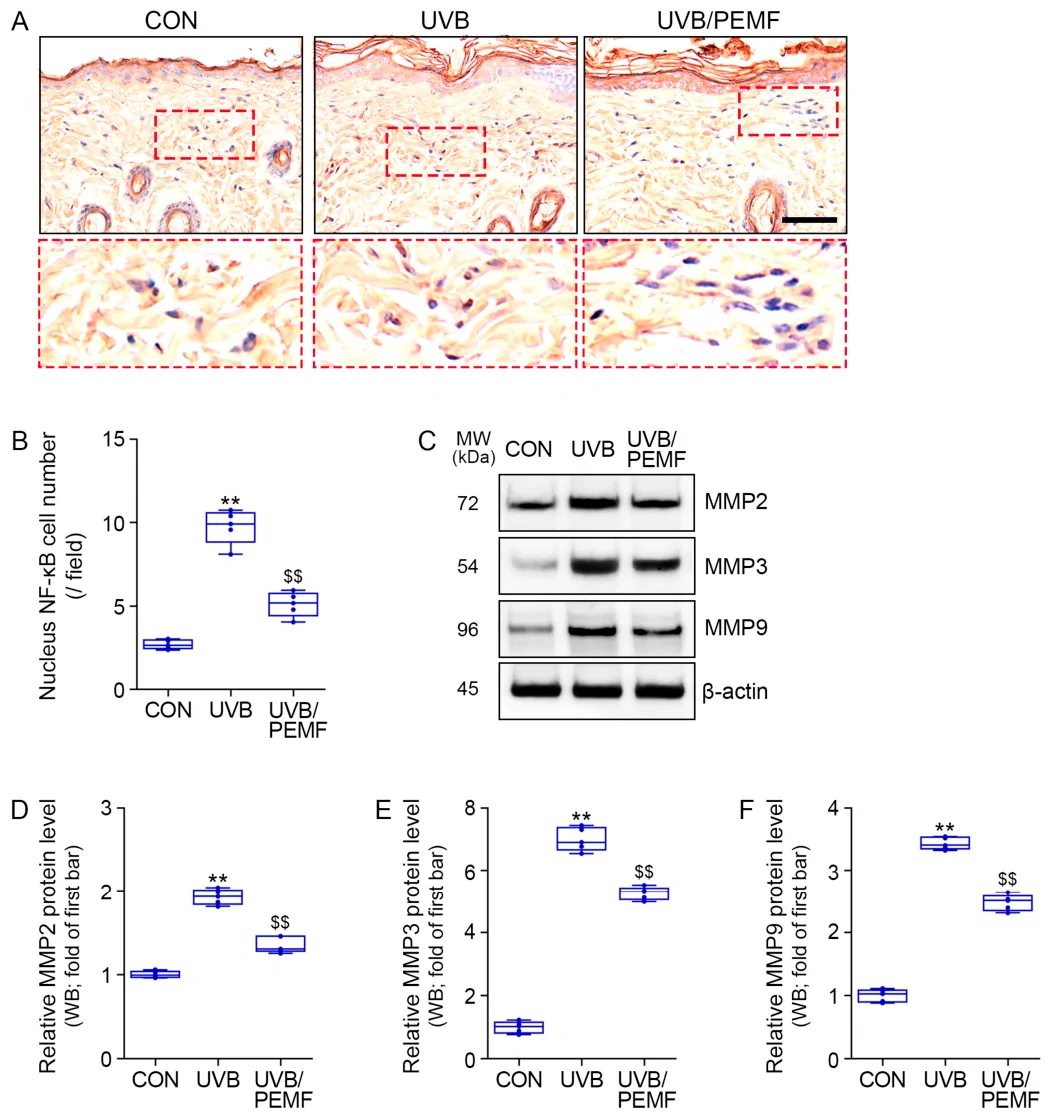
PEMF treatment suppresses NF-κB nuclear localization and MMP expression in a UVB-induced animal model. (**A**) Representative immunohistochemical images of NF-κB expression in skin tissue from the CON, UVB, and UVB/PEMF groups. The boxed regions are shown at higher magnification in the lower panels. Scale bar = 100 μm. (**B**) Quantification of cells with NF-κB-positive nuclei. (**C**) Representative Western blot images of MMP2, MMP3, MMP9, and β-actin. (**D**–**F**) Quantitative Western blot results. In all quantitative panels, groups from left to right represent CON, UVB, and UVB/PEMF. Statistical comparisons were performed using the Mann–Whitney U test. **, *p* < 0.01, vs. CON; $$, *p* < 0.01, vs. UVB. MMP, matrix metalloproteinase; PEMF, pulsed electromagnetic field.

UVB-exposed skin exhibited increased NF-κB nuclear positivity and elevated MMP2, MMP3, and MMP9 protein levels. PEMF treatment reduced NF-κB nuclear positivity and MMP expression in UVB-exposed skin (Figure 4).

**Figure 5:**
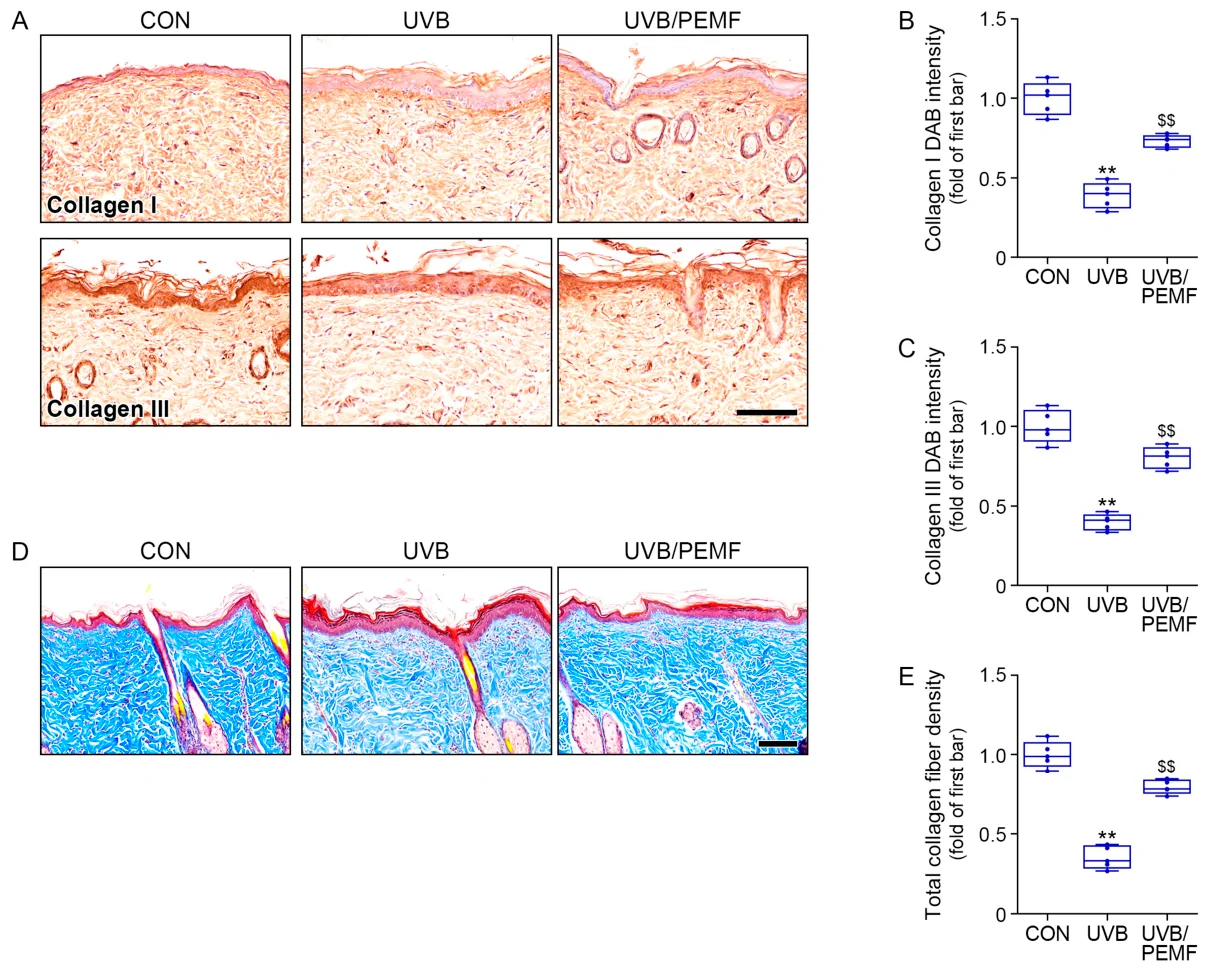
PEMF treatment improves dermal collagen-related histological features in a UVB-induced animal model. (**A**) Representative immunohistochemical images of collagen I and collagen III expression in skin tissue from the CON, UVB, and UVB/PEMF groups. Scale bar = 100 μm. (**B**,**C**) Quantitative analysis of collagen I and collagen III DAB staining intensity. (**D**) Representative Masson’s trichrome staining images showing dermal collagen fiber distribution. Scale bar = 100 μm. (**E**) Quantitative analysis of total collagen fiber density. In all quantitative panels, groups from left to right represent CON, UVB, and UVB/PEMF. Statistical comparisons were performed using the Mann–Whitney U test. **, *p* < 0.01, vs. CON; $$, *p* < 0.01, vs. UVB. PEMF, pulsed electromagnetic field.

Histological analysis showed greater collagen I and collagen III immunoreactivity and greater Masson’s trichrome-positive dermal collagen fiber density in UVB-irradiated skin treated with PEMF than UVB-irradiated skin (Figure 5).

### 2.5. PEMF Partially Attenuates UVB-Treated Epidermal Hyperplasia In Vivo

**Figure 6:**
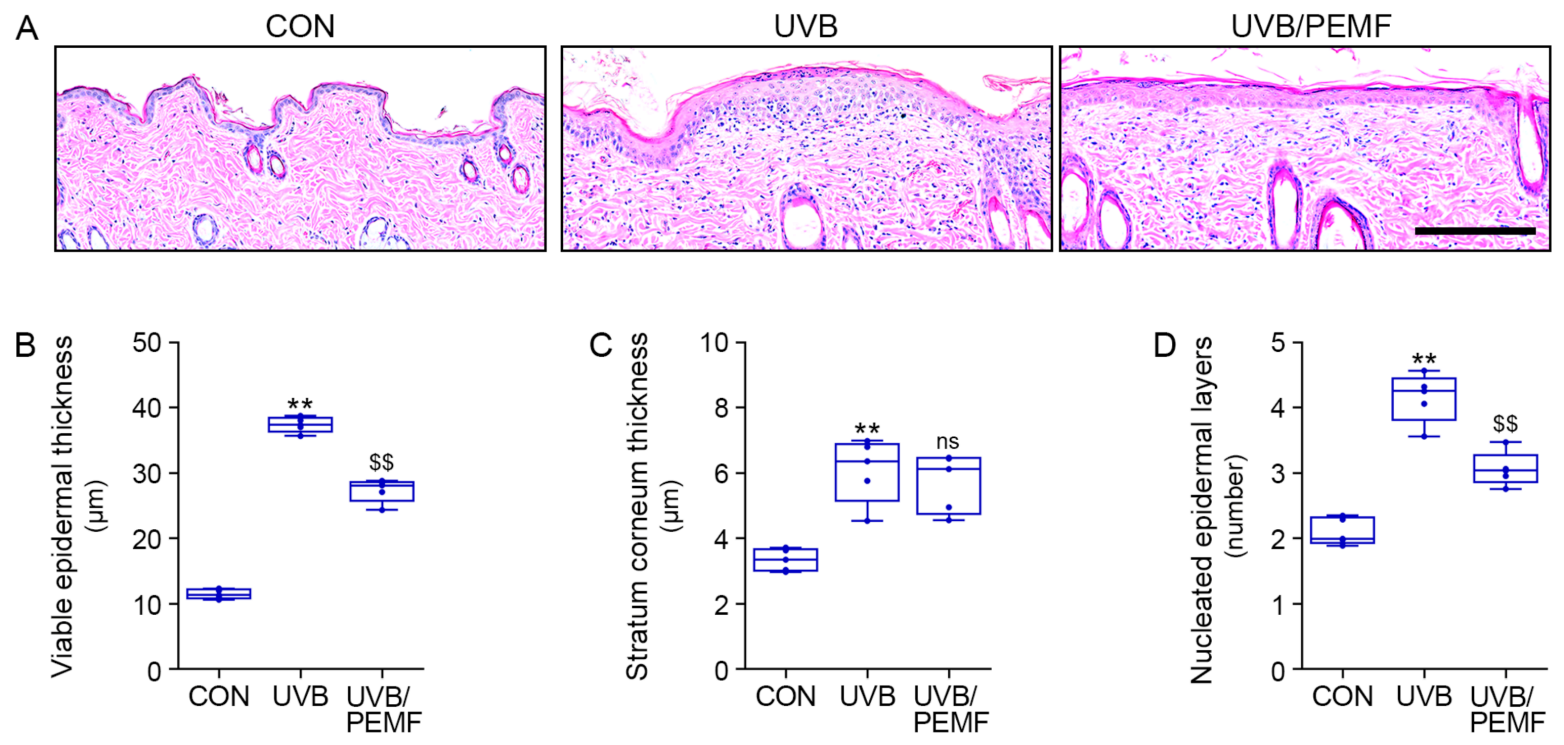
PEMF treatment partially attenuates UVB-induced epidermal hyperplasia in vivo. (**A**) Representative hematoxylin and eosin-stained skin tissue from the CON, UVB, and UVB/PEMF groups. Scale bar = 200 μm. (**B**) Viable epidermal thickness excluding the stratum corneum. (**C**) Stratum-corneum thickness. (**D**) Number of nucleated epidermal layers. In all quantitative panels, groups from left to right represent CON, UVB, and UVB/PEMF. Statistical comparisons were performed using the Mann–Whitney U test. **, *p* < 0.01, vs. CON; $$, *p* < 0.01, vs. UVB. ns, not significant; PEMF, pulsed electromagnetic field.

Hematoxylin and eosin staining demonstrated that UVB exposure increased viable epidermal thickness, stratum-corneum thickness, and the number of nucleated epidermal layers compared with untreated control skin. PEMF treatment significantly reduced viable epidermal thickness and the number of nucleated epidermal layers. In contrast, the decrease in stratum-corneum thickness after PEMF treatment was not statistically significant (Figure 6).

## 3. Discussion

In this UVB-specific experimental model, PEMF was associated with attenuation of inflammatory collagen loss. In UVB-exposed fibroblasts and animal skin, PEMF restored A2AAR, cAMP, and PKA levels and increased the pNLRP3/NLRP3 ratio. These changes were accompanied by reduced ASC/caspase-1-related inflammasome markers, lower IL-1β, decreased NF-κB nuclear positivity, reduced MMP2/MMP3/MMP9 expression, and greater collagen I, collagen III, and Masson’s trichrome-positive collagen density. The parallel response to CGS-21680 in fibroblasts is consistent with A2AAR involvement, but it does not demonstrate that A2AAR mediates the PEMF response because antagonist, knockdown, and rescue studies were not performed.

The photobiological scope of these findings requires explicit distinction between UVA and UVB. UVA penetrates into the dermis more efficiently and is strongly associated with photosensitization-derived ROS, fibroblast dysfunction, and chronic dermal matrix remodeling. UVB is absorbed predominantly in the epidermis, where it produces direct DNA photolesions, erythema, keratinocyte injury, epidermal thickening, and inflammatory mediator release; UVB can nevertheless alter dermal collagen through intercellular signaling and, in experimental systems, through direct exposure of fibroblasts [1,2,3,4]. Accordingly, our findings do not imply that UVB reproduces the full spectrum of solar photoaging or that the observed PEMF response will be identical after UVA exposure. They show that PEMF modifies inflammatory and collagen-related endpoints in a defined 306 nm UVB model.

Our results are consistent with involvement of a UVB/NLRP3/IL-1β/MMP-associated pathway. Prior keratinocyte studies show that UVB-induced nuclear and mitochondrial injury can activate NLRP3 and promote caspase-1-dependent IL-1β maturation [5,6,7]. Those keratinocyte data motivated pathway selection but do not represent evidence generated by our fibroblast experiment. In our models, UVB exposure increased inflammasome-associated and matrix-degrading markers, whereas PEMF reduced IL-1β/NF-κB/MMP-related markers and increased collagen abundance. These coordinated changes support attenuation of inflammatory matrix degradation as one plausible contributor to collagen preservation, while leaving the cellular origin and causal sequence unresolved.

PEMF has been shown to affect collagen and ECM metabolism; however, the mechanisms described in those studies differ from the pathway characterized here. The previous work demonstrates that PEMF increases collagen production in bone marrow fibroblasts by an athermal mechanism, alters collagen synthesis in skin, promotes type I collagen fiber deposition during early diabetic wound healing, enhances Col1a1, Col3a1, collagen type I, and total collagen in tendon cells, and increases disc-cell matrix synthesis through bone morphogenetic protein-dependent signaling [10,11,12,13,14]. These studies focused on repair-associated matrix accumulation, growth factor signaling, or musculoskeletal tissue regeneration [10,11,12,13,14]. In contrast, our study examined chronically UV-damaged skin and associated PEMF treatment with changes in A2AAR/cAMP/PKA/pNLRP3-related markers and suppression of inflammatory collagen-degradation pathways. This distinction is important because photoaged skin is characterized not only by reduced collagen synthesis but also by persistent collagen fragmentation and inflammatory proteolysis [8,26].

The effect of PEMF on NLRP3 is less characterized than its effects on collagen or adenosine receptors. In an osteoarthritis model, PEMF reduced NLRP3/caspase-1/gasdermin D signaling, IL-1β, and MMP-13, suggesting that PEMF-mediated inhibition of the inflammasome may suppress synovitis and cartilage degeneration [27]. However, that study did not connect PEMF-induced NLRP3 regulation to A2AAR/cAMP/PKA signaling, UV injury, or dermal collagen preservation [27]. Inflammasome studies show that cAMP/PKA signaling reduces NLRP3 inflammasome activation by limiting NLRP3-ASC interaction, ASC oligomerization, caspase-1 activation, and IL-1β secretion [21,22,23,24]. Therefore, our results integrate two previously distinct concepts—PEMF-responsive purinergic signaling and cAMP/PKA-mediated NLRP3 inhibition—in mitigating UV-induced skin photoaging.

The role for the adenosine receptor in the PEMF effect is supported by prior electromagnetic-field studies in a different biological setting. In human osteoarthritic synovial fibroblasts, electromagnetic field exposure selectively increases A2A and A3 adenosine receptor mRNA, protein expression, and receptor density, which is associated with altered cAMP responses and reduced inflammatory mediator release [18]. There is similar A2A/A3 adenosine receptor modulation in bovine chondrocytes, fibroblast-like synoviocytes, human chondrocyte lines, and osteoblasts exposed to low-frequency electromagnetic fields [19,20]. Thus, although electromagnetic fields modulate purinergic anti-inflammatory signaling, those studies primarily characterized joint-related cells rather than UV-induced matrix damage in skin cells [18,19,20]. Here, we extend the role of the adenosine receptor-mediated signaling to UV-damaged skin, showing in fibroblasts and a rat model that PEMF increases markers associated with A2AAR/cAMP/PKA/pNLRP3 signaling, attenuates IL-1β/NF-κB/MMP activation, and enhances collagen accumulation.

CGS-21680, a selective A2A adenosine receptor agonist used in adenosine receptor experiments [18,25], served as a positive control in our fibroblast experiments. CGS-21680 mimicked the effects of PEMF by restoring cAMP/PKA/pNLRP3 signaling and reducing IL-1β and collagen-degrading outputs. However, although this parallel response supports A2AAR involvement in PEMF, it does not exclude a role for A3 adenosine receptor signaling, mitochondrial effects, membrane biophysical responses, mechanosensitive channels, or other PEMF-responsive mechanisms [18,19,20,27]. Additional A2AAR antagonists such as ZM-241385, as well as A2AAR knockdown and receptor-rescue experiments in UVB/PEMF-treated models, will be required for confirmation.

Prior repair and musculoskeletal studies have suggested effects of PEMF on collagen and extracellular-matrix metabolism [10,11,12,13,14]. In the present UVB model, PEMF was associated with coordinated changes in A2AAR/cAMP/PKA/pNLRP3-related markers, inflammasome and IL-1β/NF-κB/MMP readouts, and collagen I/III abundance. These data support a perturbation-consistent model in which reduced inflammatory collagen degradation may contribute to collagen preservation. They do not establish that A2AAR is required, that the pathway is serially mediated through A2AAR/PKA/NLRP3, or that PEMF directly increases collagen synthesis.

The mechanism of PEMF action may have clinical implications. UV-induced ECM deterioration results in wrinkles, laxity, roughness, dyspigmentation, and loss of skin elasticity [28]. Treatments include photoprotection, topical retinoids, chemical peels, resurfacing lasers, intense pulsed light, radiofrequency, ultrasound-based procedures, microneedling, and combination approaches [29,30,31,32,33,34]. Based on randomized trials, topical tretinoin improves photoaging features but requires long-term adherence, which may be limited by irritation and the slow skin response [32]. Laser and light-based procedures can induce dermal remodeling but require careful consideration of wavelength, fluence, pulse duration, density, and skin phototype. Further, these procedures may cause discomfort, erythema, edema, crusting, infection, dyspigmentation, post-inflammatory hyperpigmentation, scarring, and downtime [29,30,31,33,34]. PEMF is non-optical and does not create chromophore-dependent photothermal injury; therefore, it may serve as an adjunct or maintenance therapy by targeting the inflammatory component of ECM degradation. However, there is no evidence for the clinical superiority of PEMF over topical agents, lasers, radiofrequency, ultrasound, or microneedling, and comparative studies in humans are required to determine any therapeutic advantage.

The H&E analysis was performed to determine whether the dermal collagen-preserving response to PEMF was accompanied by exacerbation of UVB-induced epidermal injury. UVB increased viable epidermal thickness, stratum-corneum thickness, and the number of nucleated epidermal layers. PEMF did not further increase any of these measures; instead, it significantly reduced viable epidermal thickness and the number of nucleated epidermal layers, although the reduction in stratum-corneum thickness was not statistically significant. Within the measured values, these findings provide no evidence that PEMF aggravated the burn-like epidermal changes while preserving dermal collagen. However, because keratinocyte apoptosis, barrier function, and standardized composite injury scores were not assessed, the findings should not be interpreted as evidence of complete epidermal protection.

Several limitations should be acknowledged. First, the study used a 306 nm UVB model. UVA, which penetrates more deeply into the dermis and is a major driver of ROS-mediated chronic dermal photoaging, was not tested; therefore, these findings should not be generalized to the full solar UV spectrum or to UVA-dominant photoaging. Second, the cell experiments used fibroblasts only. Keratinocyte evidence was used to motivate the NLRP3 pathway, but epidermal–dermal crosstalk, keratinocyte injury, melanocyte responses, macrophages, and barrier function were not experimentally evaluated. Third, H&E morphometry quantified viable epidermal thickness, stratum-corneum thickness, and nucleated epidermal layers; however, keratinocyte apoptosis, barrier-function markers, transepidermal water loss, and standardized composite injury scores were not evaluated. The present data therefore support partial attenuation of epidermal hyperplasia but not complete protection from UVB-induced epidermal injury. Fourth, A2AAR involvement was inferred from pathway co-changes and CGS-21680 mimicry, not demonstrated by A2AAR antagonism, knockdown, or receptor-specific rescue. Fifth, PKA dependency and the functional role of NLRP3 Ser295 phosphorylation were not tested with PKA inhibitors or NLRP3 phospho-mutants, and canonical inflammasome assembly was not assessed using NLRP3-ASC interaction or ASC oligomerization assay. Sixth, collagen outcomes reflected collagen I/III abundance and histological density rather than direct synthesis and turnover measurements. Finally, the small animal study, absence of sham PEMF exposure and dose–response optimization, and lack of aged human skin, phototype-stratified safety, and clinical comparators limit translation.

## 4. Materials and Methods

### 4.1. PEMF Treatment

PEMF (Corefacial; K1MED GLOBAL, Seoul, Republic of Korea) treatment used a PEMF-generating device to deliver time-varying electromagnetic pulses to cultured fibroblasts or animal skin tissue. The PEMF exposure parameters were a frequency of 20 Hz, magnetic flux density of 149 mT, pulse duration of 220 μs, and exposure time of 3 min. Each sample or animal received a single PEMF exposure.

For in vitro experiments, culture plates were placed in the exposure area of the PEMF device under standard culture conditions. For in vivo experiments, PEMF stimulation was applied to UVB-exposed skin of animals. To minimize handling-related bias, the untreated-control and UVB-control groups received the same handling, environmental conditions, and procedures, except for active PEMF exposure.

### 4.2. In Vitro Study

Human dermal fibroblasts (HDFs; CEFO Co., Ltd., Seoul, Republic of Korea) were cultured in CEFOgro^TM^ Human MSC Growth Medium (CEFO Co., Ltd.) at 37 °C in a humidified atmosphere containing 5% CO_2_. Cells were seeded at an appropriate density depending on each assay and stabilized for 24 h before UVB irradiation. Cells were washed with phosphate-buffered saline before irradiation with G15T8E lamps (Sankyo Denki Co., Ltd., Hiratsuka, Japan; peak wavelength, 306 nm). Based on the manufacturer-reported output and experimental geometry, the estimated UVB dose was approximately 10 mJ/cm^2^ per exposure. After irradiation, the cells received fresh medium and were incubated for 24 h. Cells were subsequently treated with PEMF or the A2AAR agonist CGS-21680 (Sigma-Aldrich, St. Louis, MO, USA) at 1 μM [35]. After 48 h, culture supernatants or cell lysates were collected. The groups were (1) CON, (2) UVB, (3) UVB/PEMF, and (4) UVB/CGS-21680 (Figure S1).

### 4.3. In Vivo Experiments

Male Sprague–Dawley rats (8 weeks old) purchased from Orient Bio (Seongnam, Republic of Korea) were acclimatized for 7 days and housed at 22 ± 2 °C and 50–60% humidity under a 12-h light/dark cycle with free access to food and water. Animals were randomly allocated to three groups (*n* = 5 per group): CON, UVB, and UVB/PEMF. No animals were excluded from the study or statistical analysis.

All animal procedures were conducted in accordance with institutional guidelines and were approved by the Institutional Animal Care and Use Committee of Gachon University (IACUC; LCDI-2025-0086). This study complied with the ethical standards of the Association for Assessment and Accreditation of Laboratory Animal Care International (AAALAC International, Frederick, MD, USA) and adhered to the Animal Research: Reporting of In Vivo Experiments (ARRIVE) guidelines. Each rat was treated and analyzed individually throughout the study. No humane endpoints were reached during the study.

To induce UVB-mediated skin damage, rats were irradiated with a G15T8E UVB lamp (Sankyo Denki Co., Ltd., peak wavelength, 306 nm) positioned approximately 50 cm above the skin. UVB irradiation was applied once every 2 days for 30 days (15 exposures). Based on the manufacturer-reported output and experimental geometry, the estimated dose was approximately 300 mJ/cm^2^ per exposure [36]. After UVB-induced skin damage was established, the UVB/PEMF group received PEMF treatment on Day 31. Subsequently, UVB irradiation was applied once every 2 days for 28 days (14 additional exposures). During device application, rats were anesthetized with inhaled isoflurane at an induction concentration of 3% and a maintenance concentration of 1.5%, with oxygen supplied at a flow rate of 0.5 L/min. On Day 59, rats were euthanized by CO_2_ exposure in a chamber for at least 3 min. Skin was collected for biochemical, molecular, and histological analyses (Figure S2).

### 4.4. Sample Preparation

For cell-based experiments, culture supernatants were collected for the enzyme-linked immunosorbent assay (ELISA) of secreted IL-1β. Cells were lysed using RIPA lysis buffer (ATTO Corporation, Tokyo, Japan) containing protease and phosphatase inhibitors for protein extraction, ELISA, cAMP assay, and Western blot analysis. For immunocytochemistry, cells were fixed with 4% paraformaldehyde and processed for staining.

For animal experiments, harvested skin tissues were divided into two portions. One portion was immediately frozen and stored at −80 °C until protein extraction and the other portion was fixed in 4% paraformaldehyde, embedded in paraffin, and sectioned at 7 μm thickness for histological and immunohistochemical analyses.

### 4.5. Enzyme-Linked Immunosorbent Assay

Equal amounts of protein extracts from cultured cells or skin tissues were coated onto 96-well plates in 0.05 M carbonate–bicarbonate buffer and incubated overnight at 4 °C. After washing with phosphate-buffered saline containing 0.1% Tween-20 (PBS-T), the plates were blocked with 5% skim milk and incubated with primary antibodies (Table S1) overnight at 4 °C, followed by horseradish peroxidase (HRP)-conjugated secondary antibodies (Vector Laboratories, Burlingame, CA, USA). Color development was performed using tetramethylbenzidine (TMB) substrate solution (Sigma-Aldrich), and the reaction was terminated with 2 N sulfuric acid. Absorbance was measured at 450 nm using a microplate reader. The results were expressed as fold changes relative to the control group. For IL-1β secretion analysis, culture supernatants were analyzed using the same procedure.

### 4.6. Cyclic AMP (cAMP) Assay

Intracellular cAMP levels were measured using the Cyclic AMP XP^®^ Assay Kit (Cell Signaling Technology, Danvers, MA, USA) according to the manufacturer’s instructions. cAMP levels were calculated according to the manufacturer’s instructions and expressed as fold changes relative to the control group.

### 4.7. Western Blot Analysis

Equal amounts of protein were separated by SDS-PAGE and transferred onto Immobilon^®^-P polyvinylidene difluoride (PVDF) membranes (Millipore, Billerica, MA, USA) using an EzFastBlot transfer system (ATTO Corporation, Tokyo, Japan). Membranes were blocked with 5% skim milk in Tris-buffered saline containing 0.1% Tween-20 (TBS-T) and incubated with primary antibodies (Table S1). After washing with TBS-T, membranes were incubated with HRP-conjugated secondary antibodies (Vector Laboratories). Protein bands were visualized using Amersham™ ECL Select (Cytiva, Marlborough, MA, USA) and imaged using a ChemiDoc™ Imaging System (Bio-Rad Laboratories, Hercules, CA, USA). Band intensities were quantified using ImageJ software (version 1.53s; National Institutes of Health, Bethesda, MD, USA) and normalized to β-actin.

### 4.8. Immunocytochemistry and Immunohistochemistry

For immunocytochemistry, cultured cells were fixed with 4% paraformaldehyde, permeabilized with 0.1% Triton X-100, and blocked with 5% normal serum. Cells were incubated with primary antibodies (Table S1) overnight at 4 °C, followed by Alexa Fluor™ 488-conjugated secondary antibodies (Invitrogen, Carlsbad, CA, USA). Nuclei were counterstained with DAPI (Sigma-Aldrich).

For immunohistochemistry, paraffin-embedded skin sections were deparaffinized, rehydrated, and subjected to heat-induced antigen retrieval in 10 mM citrate buffer (pH 6.0). Endogenous peroxidase activity was then blocked with 3% hydrogen peroxide. Sections were blocked with 5% normal serum, incubated with primary antibodies (Table S1), followed by biotinylated secondary antibodies (Vector Laboratories). Sections were then incubated with the VECTASTAIN^®^ Elite ABC reagent (Vector Laboratories). Immunoreactivity was visualized using 3,3′-diaminobenzidine (DAB; Sigma-Aldrich) and counterstained with hematoxylin (KPNT, Cheongju, Republic of Korea).

Images were acquired using a VS200 Slide Scanner (Olympus Corporation, Tokyo, Japan). Five randomly selected images per sample were analyzed [37]. Positive staining areas were quantified using ImageJ software (version 1.53s; National Institutes of Health, Bethesda, MD, USA).

### 4.9. Masson’s Trichrome Staining

To determine dermal collagen fiber density, paraffin-embedded skin sections were stained with a Masson’s Trichrome Stain Kit (ScyTek Laboratories, Logan, UT, USA) according to the manufacturer’s instructions. Images were acquired using a VS200 Slide Scanner (Olympus Corporation), and five randomly selected images per sample were analyzed [38]. Collagen-positive areas were quantified using ImageJ software (version 1.53s; National Institutes of Health).

### 4.10. Hematoxylin and Eosin Staining and Epidermal Morphometric Analysis

To evaluate epidermal morphology and UV-induced epidermal alterations, paraffin-embedded skin sections were stained with hematoxylin and eosin (H&E) according to standard procedures (KPNT, Cheongju, Republic of Korea). Images were acquired using a VS200 Slide Scanner (Olympus Corporation), and five randomly selected images per sample were analyzed and quantified using ImageJ software (version 1.53s; National Institutes of Health). Viable epidermal thickness was measured perpendicular to the skin surface from the basement membrane to the upper boundary of the stratum granulosum, excluding the stratum corneum. Stratum corneum thickness was measured separately from the upper boundary of the viable epidermis to the outermost surface of the stratum corneum. The number of nucleated epidermal cell layers was counted along a perpendicular line extending from the basal layer to the uppermost nucleated epidermal layer, avoiding hair follicles and areas with sectioning artifacts.

### 4.11. Statistical Analysis

Sample size was determined based on previous studies using similar experimental models and practical considerations. Outcome assessment and data analysis were performed in a blinded manner where possible. Statistical analyses were performed using SPSS version 26 (IBM Corp., Armonk, NY, USA). Data normality was assessed using the Shapiro–Wilk test. Although no significant deviations from normality were detected, nonparametric statistical methods were used because the small sample size in each group (*n* = 5) limits the power of normality tests to detect departures from normality [39,40]. Prespecified pairwise comparisons were performed using the Mann–Whitney U test. Data are presented as box-and-whisker plots showing the median, interquartile range, minimum and maximum values, and individual data points. Statistical significance is indicated in the figure legends.

## 5. Conclusions

PEMF exposure using Corefacial attenuated inflammatory matrix degradation and preserved collagen-related endpoints in fibroblast and animal models subjected to 306 nm UVB. The coordinated changes in A2AAR/cAMP/PKA/pNLRP3-related markers, inflammasome-associated readout, IL-1β/NF-κB/MMP signaling, and collagen abundance are consistent with involvement of this signaling network, but receptor-specific mediation and direct collagen synthesis were not established. Because UVA, keratinocytes, epidermal injury endpoints, and human clinical outcomes were not tested, the findings should be interpreted as preclinical evidence from a UVB-specific model.

## Data Availability

The original contributions presented in this study are included in the article/Supplementary Materials. Further inquiries can be directed to the corresponding authors.

## Supplementary Materials

The following supporting information can be downloaded at https://www.mdpi.com/article/10.3390/ijms27177904/s1.
