# Supplementary material for: Pulsed Electromagnetic Field Exposure Attenuates Ultraviolet B-Induced Dermal Collagen Loss in Association with A2A Adenosine Receptor Signaling
Source: Int J Mol Sci. 2026 Sep 4;27(17):7904. doi: 10.3390/ijms27177904 (PMC13566763; doi:10.3390/ijms27177904)
Supplement: Supplementary file 1 [file ijms-27-07904-s001.zip › ijms-4503700-supplementary.pdf]

International Journal of
Molecular Sciences

Supplementary Materials

Pulsed Electromagnetic Field Exposure Attenuates Ultraviolet B-Induced Dermal Collagen Loss in Association with A2A Adenosine Receptor Signaling

Kyung-A Byun [1,2,3,†], Jae Ik Lee [4,†], Seyeon Oh [2,3], So Eun Kim [5], Suk Bae Seo [6], Kuk Hui Son [7,*] and Kyunghee Byun [1,3,8,*]

1 Department of Anatomy & Cell Biology, College of Medicine, Gachon University, Incheon 21936, Republic of Korea
2 LIBON Inc., Incheon 22006, Republic of Korea
3 Functional Cellular Networks Laboratory, Lee Gil Ya Cancer and Diabetes Institute, Gachon University, Incheon 21999, Republic of Korea
4 Department of Thoracic and Cardiovascular Surgery, International Healthcare Center, Seoul National University Bundang Hospital, Seongnam 13620, Republic of Korea
5 Cheongdam Ruby Clinic, Seoul 06009, Republic of Korea
6 SeoAh Song Dermatologic Clinic, Seoul 05557, Republic of Korea
7 Department of Thoracic and Cardiovascular Surgery, Gachon University Gil Medical Center, Gachon University, Incheon 21565, Republic of Korea
8 Department of Health Sciences and Technology, Gachon Advanced Institute for Health & Sciences and Technology (GAIHST), Gachon University, Incheon 21999, Republic of Korea
* Correspondence: dr632@gachon.ac.kr (K.H.S.); khbyun1@gachon.ac.kr (K.B.); Tel.: +82-32-460-3666 (K.H.S.); +82-32-899-6511 (K.B.)
† These authors contributed equally to this work.

Table S1. List of antibodies used in this study.

Antibody	Company	Dilution rate		
		ELISA	Western blot	ICC/IHC
A2AAR	Novus Biologicals	1:500	-	-
PKA	Santa Cruz Biotechnology	1:500	-	-
IL-1β	Bioss	1:500	-	-
COL1	Santa Cruz Biotechnology	1:500	-	-
COL3	Santa Cruz Biotechnology	1:500	-	-
β-actin	Cell Signaling Technology	-	1:1,000	-
pNLRP3		-	1:1,000	-
NLRP3	BOSTER	-	1:1,000	-
ASC	Santa Cruz Biotechnology	-	1:1,000	-
Caspase 1	Santa Cruz Biotechnology	-	1:1,000	-
MMP2	GeneTex	-	1:1,000	-
MMP3	ABclonal	-	1:1,000	-
MMP9	ABclonal	-	1:1,000	-
NF-κB	Cell Signaling Technology	-	-	1:100

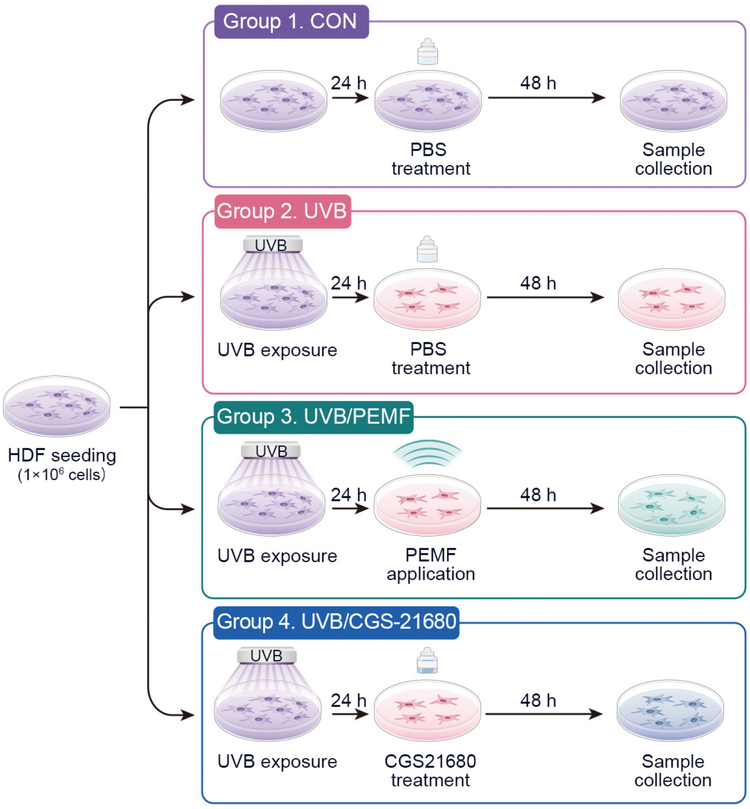

Figure S1. Schematic illustration of the *in vitro* experimental design. Human dermal fibroblasts (HDFs) were stabilized for 24 h, exposed to UVB irradiation, and then treated with PEMF or the A2A adenosine receptor agonist CGS-21680. The experimental groups were CON, UVB, UVB/PEMF, and UVB/CGS-21680.

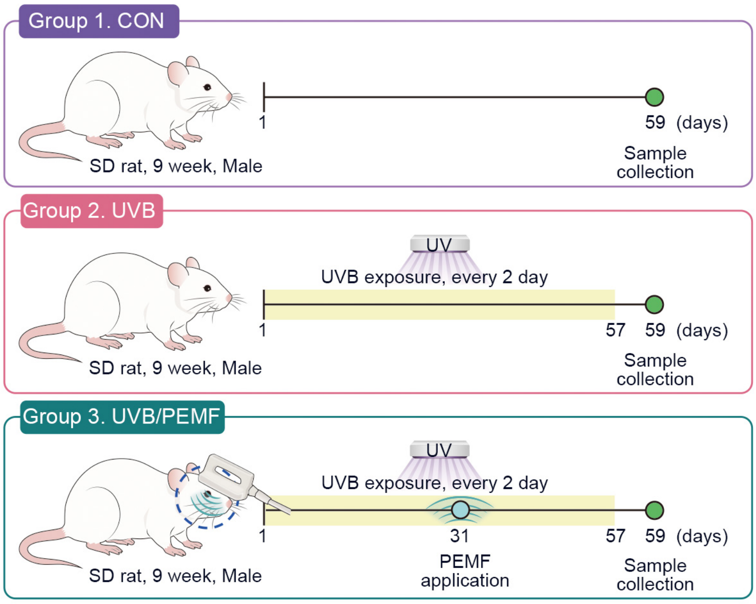

Figure S2. Schematic illustration of the *in vivo* experimental design. PEMF treatment was applied to the UVB-exposed facial skin region of 9-week-old, male, Sprague–Dawley (SD) rats after UVB-induced skin damage was established. The experimental groups were CON, UVB, and UVB/PEMF.
